# Surface functionalization of graphene nanosheet with poly (l-histidine) and its application in drug delivery: covalent vs non-covalent approaches

**DOI:** 10.1038/s41598-022-21619-0

**Published:** 2022-11-09

**Authors:** Zahra Najafi rad, Farzaneh Farzad, Leila Razavi

**Affiliations:** Department of Chemistry, University of Birjand, Birjand, Iran

**Keywords:** Cancer, Drug discovery

## Abstract

Nowadays, nanomaterials are increasingly being used as drug carriers in the treatment of different types of cancers. As a result, these applications make them attractive to researchers dealing with diagnosis and biomarkers discovery of the disease. In this study, the adsorption behavior of gemcitabine (GMC) on graphene nanosheet (GNS), in the presence and absence of Poly (L-histidine) (PLH) polymer is discussed using molecular dynamics (MD) simulation. The MD results revealed an increase in the efficiency and targeting of the drug when the polymer is covalently attached to the graphene substrate. In addition, the metadynamics simulation to investigate the effects of PLH on the adsorption capacity of the GNS, and explore the adsorption/desorption process of GMC on pristine and PLH- grafted GNS is performed. The metadynamics calculations showed that the amount of free energy of the drug in acidic conditions is higher (− 281.26 kJ/mol) than the free energy in neutral conditions (− 346.24 kJ/mol). Consequently, the PLH polymer may not only help drug adsorption but can also help in drug desorption in lower pH environments. Based on these findings, it can be said that covalent polymer bonding not only can help in the formation of a targeted drug delivery system but also can increase the adsorption capacity of the substrate.

## Introduction

Gemcitabine (GMC) anti-cancer drug is used extensively to treat a variety of cancers such as pancreatic, breast, and lung cancer, etc.^1,2^. GMC as a prodrug enters the cell through nucleoside transporters and blocks cell growth by inhibiting the DNA synthesis in cells and leading to apoptosis of the cancer cells^3^. However, like the other anti-cancer drugs, this drug has a lot of side effects, such as bone marrow failure, liver and kidney problems, mouth ulcers, and neuropathy^4^. These disadvantages necessitate the design and development of an appropriate drug delivery system (DDS) for its delivery to target tissues. Many researches have shown that DDSs can increase the effectiveness of anti-cancer drugs and have beneficial therapeutic effects on cancer treatment^5–7^. Nowadays, in various drug formulation ns such as dermatological, oral, injectable, etc., there is no control over the speed, timing, and location of drugs^8^. Hence, the use of DDSs is essential to increase the bioavailability of the drugs at particular sites in the body and at specific times. The advantages of drug DDSs include maintaining delivering multiple drugs with one formulation, the concentration of the drug for a specified period, the ability to deliver the drug to the specific site, and adjusting the rate of the drug release at the target tissue^9^. These systems reduce the toxicity and side effects of the drugs on healthy organs and increase the therapeutic effects by delivering the drug to the desired location^10^. Besides, in the DDSs, the biological and physical properties of the drug are preserved until it achieves the desired tissue. Hitherto, various nanomaterials with different shapes, sizes, and chemical properties have been studied as carriers to deliver therapeutic factors. These nano-carriers include dendrimers, micelles, polymer matrixes, liposomes, metals, metal oxides, and carbon nanomaterials (e.g., carbon nanotubes, fullerenes, and, graphene nanosheet (GNS))^11–14^.

Among the nano-carriers, GNS has shown great potential for utilization in targeted therapeutics. As a two-dimensional (2D) carbon nanosheet, GNS is made of sp^2^-hybridized C atoms and is packed in a honeycomb lattice^15–19^. In fact, GNS comprises some layers with π-conjugated six-membered rings that composes an aromatic and planar macromolecule. The planar structure supplies an extraordinary ability to immobilize various compounds such as drug molecules, biomolecules, fluorescent cells, and metals^20^. Given that pristine GNS is very hydrophobic and poorly water-soluble, it's better using of surface modifiers for its application in pharmacology and biomedicine^21–23^. GNS has shown a loading capacity of 200%, which is significantly superior to other DDSs^24,25^. As a result, GNS that has been correctly modified can act as an appropriate material for the delivery of genes and anticancer drugs, biosensors, bioimaging, tissue engineering, antimicrobial application, and cell culture^26–30^.

Poly(l-histidine) (PLH) is one of the most prevalent pH-responsive polypeptides which undergoes a solubility transition within the physiological and tumor tissue pH and possesses a pK_a_ of about 6.5 due to its imidazole ring. Moreover, PLH, like the other cationic polymers, can be caused endosomal escape due to the high pH-buffering capacity of this biomacromolecule^31,32^. Therefore, the composition of PLH and GNS will provide appropriate ground for designing advanced DDSs. Lately, Zhang et al.^33^ carried out a series of computational and experimental researches on the interactions of doxorubicin with GNS. They reported that the GNS-histidine complex is stabilized by π-π interactions. Also, their results illustrated that functionalized GNS not only reveals high biocompatibility with cells in vitro but also increases the adsorption of antitumor drugs by cells.

Herein, firstly the dynamic adsorption process of GMC molecule on the surfaces of pristine GNS and GNS functionalized (F-GNS) with PLH, as covalently and non-covalent modification, via molecular dynamics (MD) simulation will be studied^34,35^. Afterward, we performed a well-tempered metadynamics (WT-MD) simulation^36,37^ to elucidate the effects of PLH on the adsorption capacity of GNS in both neutral and acidic environments.

## Materials and methods

### Molecular models and initial structures

**Figure 1:**
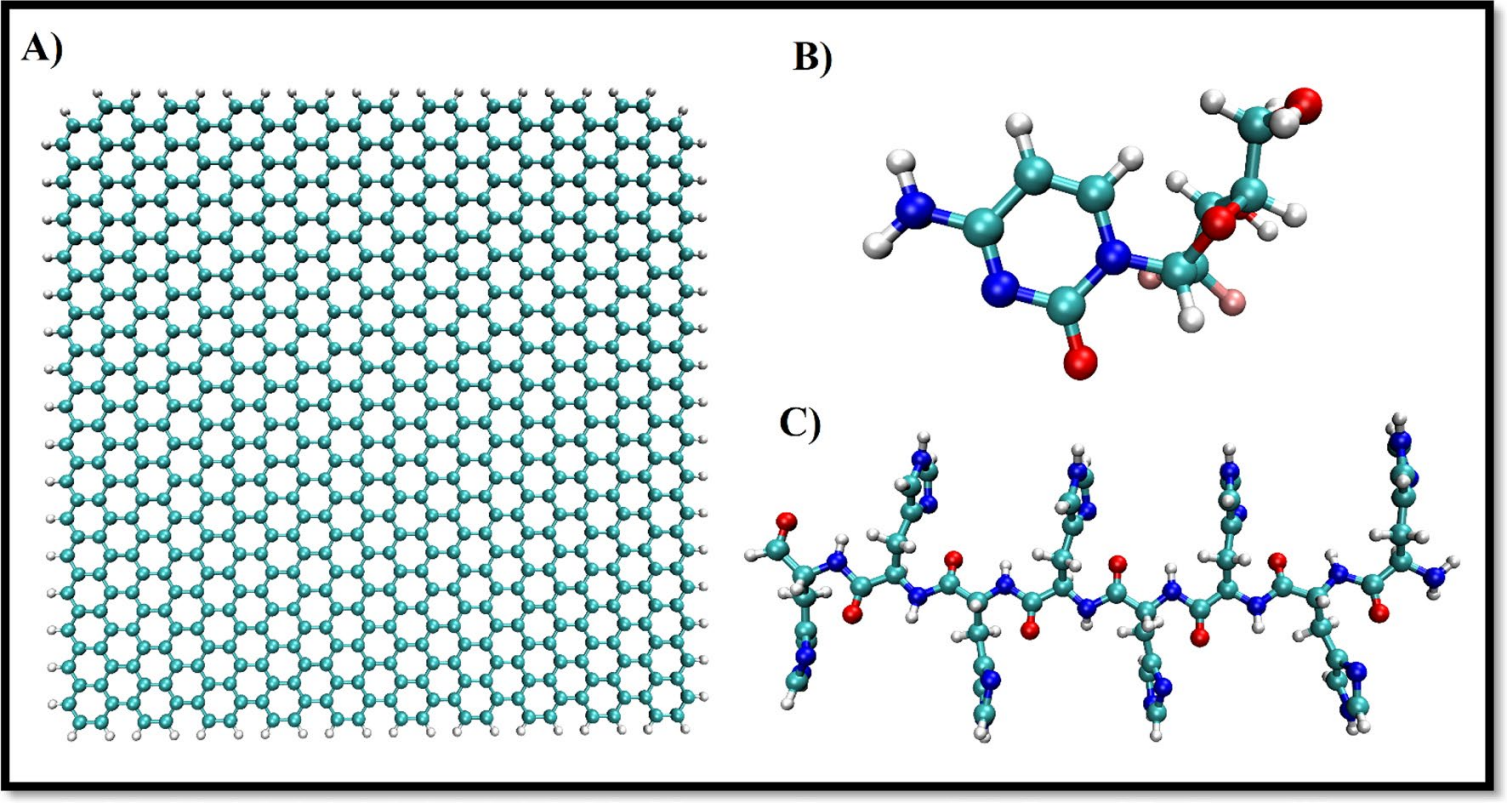
The structure of (**A**) graphene nanosheet, (**B**) gemcitabine drug, and (**C**) PLH polymer, respectively.

The initial structure of the GNS (40*40 Å^2^), including 678 carbon atoms and 72 hydrogen atoms, is acquired via the Gauss view program^38^. The edges are saturated with hydrogen atoms to compensate for the dangling bonds. GMC molecule is given from the PubChem database (PubChem CID: 60750). PLH molecule is modeled via Gauss view 06 software, and its topology file is implemented using the CHARMM36 force field^39^. Figure 1 shows the chemical structure of the GNS carrier, GMC molecule, and PLH polymer.

### Force fields and simulation boxes

Force field parameters for the nanocarrier, drug, and polymer are generated with the CHARMM general force field. Overall, three simulation systems are designed, and MD simulation is carried out, which are afforded in Table 1. In the GNS_PLH/GMC system, four chains of PLH (each chain contains eight-unit monomers) as covalently attached to the nano-carrier.

**Table 1:** Details of the MD simulation systems.

Systems	Constituents	No. adsorbed	No. Water molecules	No. Na^+^	No. Cl^-^	Box size (nm^3^)
GRA/GEM	GNS + GMC	4	16,489	47	46	8 × 8 × 8
GRA_PLH/GEM	GNS + PLH (covalently) + GMC	7	18,498	47	49	8 × 8 × 8
GRA-PLH/GEM	GNS + PLH (non-covalent) + GMC	5	16,290	46	59	8 × 8 × 8

For the GNS-PLH/GMC system, the final orientation of GMC on GNS is extracted and then noncovalently functionalized with PLH chains. In each of the simulation systems, ten GMC molecules are located about 2 nm away from the carrier surface (see Fig. S1).

### MD simulation

Trajectory analysis is performed using programs within the GROMACS 5.1.4 (ver 2019.2) software^40,41^. The temperature (310 K, normal body temperature) and pressure (1 bar) are kept constant using the V-rescale and Berendsen algorithms, respectively^42,43^. All production MD runs under periodic boundary circumstances are carried out for 60 ns in each system. All bonds at their equilibrium length are constrained with the LINCS algorithm^44^. A 1.4 nm cut-off is adopted for van der Waals interactions, while the long-range electrostatic interactions are examined based on the Particle-mesh Ewald Method^45^. The visual molecular dynamics package is utilized to view the entire simulation process^46^.

#### MD simulation: evaluation methods

A set of descriptors are derived for investigation of the simulation outputs, which are briefly outlined below:

##### Radial distribution function (RDF)

The RDF is calculated by Rog and Martinez-Seara's methodology to examine the distributions of drug molecules around the carrier^46^. Indeed, the analysis of RDF data shows how the particles in a system are arranged radially. RDF, or pair correlation function g_ij_(r), between particles of type i and j, is defined as the following equation:

1 $${\text{g}}_{{{\text{ij}}}} \left( {\text{r}} \right) = \frac{{\left\langle {{\uprho }_{j } \left( r \right)} \right\rangle }}{{\left\langle {{\uprho }_{j } } \right\rangle_{local } }}$$

where, $<{\uprho }_{j }\left(r\right)>$ as the particle density of type j at a distance r around particles i is defined, and ${{<\uprho }_{j }>}_{local}$ is the particle density of type j averaged over all spheres around particles i with radius r_max_.

##### The mean square displacement (MSD)

For the designation of diffusivity of GMC molecules adsorbed to GNSs in different systems, at first, the following statement is used to estimate the MSD^47,48^:

2 $${\text{MSD }}\left( {\Delta {\text{t}}} \right) \, = \, \left\langle {\left( {{\text{r}}_{{\text{i}}} \left( {\Delta {\text{t}}} \right) \, - {\text{r}}_{{\text{i}}} \left( 0 \right)} \right)^{{2}} } \right\rangle \, = \, \left\langle { \, \Delta {\text{r}}_{{\text{i}}} \left( {\Delta {\text{t}}} \right)^{{2}} } \right\rangle$$

During the simulation time, particle diffusion coefficient (D_i_) can be calculated from Einstein's equation, as expressed in Eq. (2).

3 $${\text{D}}_{{\text{i}}} = \frac{1}{6}{\Delta t}\mathop {\lim }\limits_{{{\Delta t} \to \infty }} {\text{MSD }}\left( {{\Delta t}} \right)$$

##### The number of hydrogen bonds (HB)

According to the geometric criteria, as follows, HB analyses examine the number of possible HBs made between donors and acceptors^49^.

4 $${\text{r}} \le {\text{r}}_{HB} = 0.35\,\,{\text{nm}}\,\,{\text{and}}\,\,\alpha \le \alpha_{HB} = 30^{ \circ }$$

The number of HBs made between the simulated components over time provides valuable insight into the nature of the forces between them.

### The WT-MD calculation

To better understand the differences between the interactions of GMC with F-GNS in the neutral and acidic environment, two series of WT-MD simulations are performed. For each case, a simulation box with a 3.5 × 3.5 × 8 Å^3^ cubic dimension is designed that is contained a GMC on one side and a PLH on the other side. To construct protonated systems, the final structure of the GNS-PLH/GMC system is extracted, and then the relevant imidazole ring of PLH is protonated^50^. To create pH level for the GNS-PLH/GMC system, the technique proposed by Adnan et al. is used in this work^51^. The sum_hills tool in Gromacs 2019.2^52,53^ patched with PLUMED version 2.5.2 plugin^54^ is utilized to do WT-MD simulations. The free energy (FE) surface of the investigated systems as a function of the COM’s GMC from the PLH (collective variable CV_COM_) is computed. This CV allows the GMC comes close to the polymer in different orientations and conformations. For two studied complexes, the WT-MD simulation is run for 120 ns.

## Results and discussions

### MD simulation

#### Drug loading on the GNS and F-GNSs

One of the most significant applications of 2D nanomaterials in drug delivery is their ability to carry a high dose of drugs that can be affected by various agents^55^. In the present research, to examine the effect of the PLH functional group on the GMC adsorption mechanism, a high number of GMC and PLH (relative to the size of the selected GNS) is utilized.

**Figure 2:**
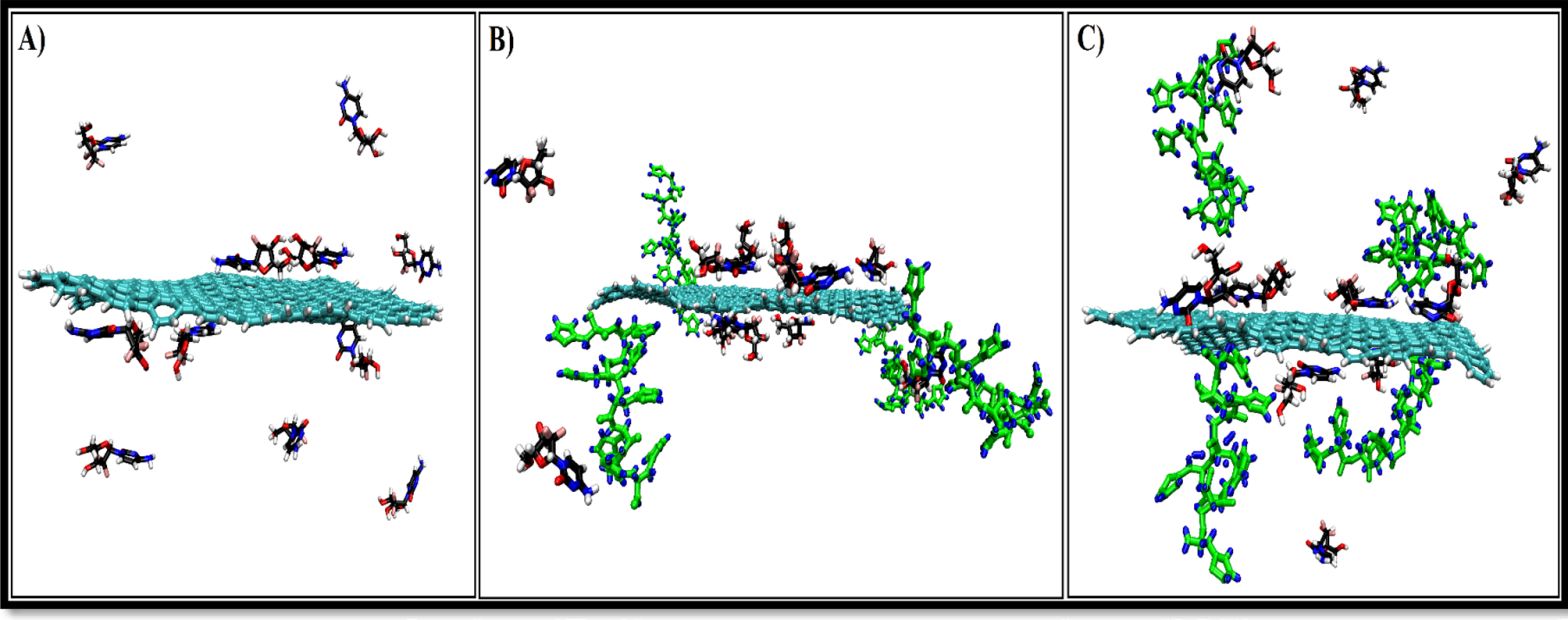
The final snapshots of (**A**) GNS/GMC, (**B**) GNS_PLH/GMC, and (**C**) GNS-PLH/GMC systems. The number of adsorbed GEM molecules in the GNS/GMC, GNS_PLH/GMC and GNS-PLH/GMCsystems are four, seven and five drug molecules, respectively.

The initial and final snapshots for the GMC adsorption on the pristine and functionalized GNSs are shown in Figs. S1 and S2. The final snapshots in Fig. 2 show that most of the GMC molecules adsorbed on the F-GNS surface after 60 ns in functionalized systems, while the number of drugs adsorbed in the pristine system is less. These findings can be attributed to the presence of the PLH functional group, which increases the tendency of the drug molecules to adsorb on F-GNS surfaces. The drug molecules interact with the nanocarrier through the formation of H-bond and π-π interactions. In addition, the final snapshots show that drug uptake in the GNS_PLH/GMC system is better than the other two systems.

#### Interaction energies

**Figure 3:**
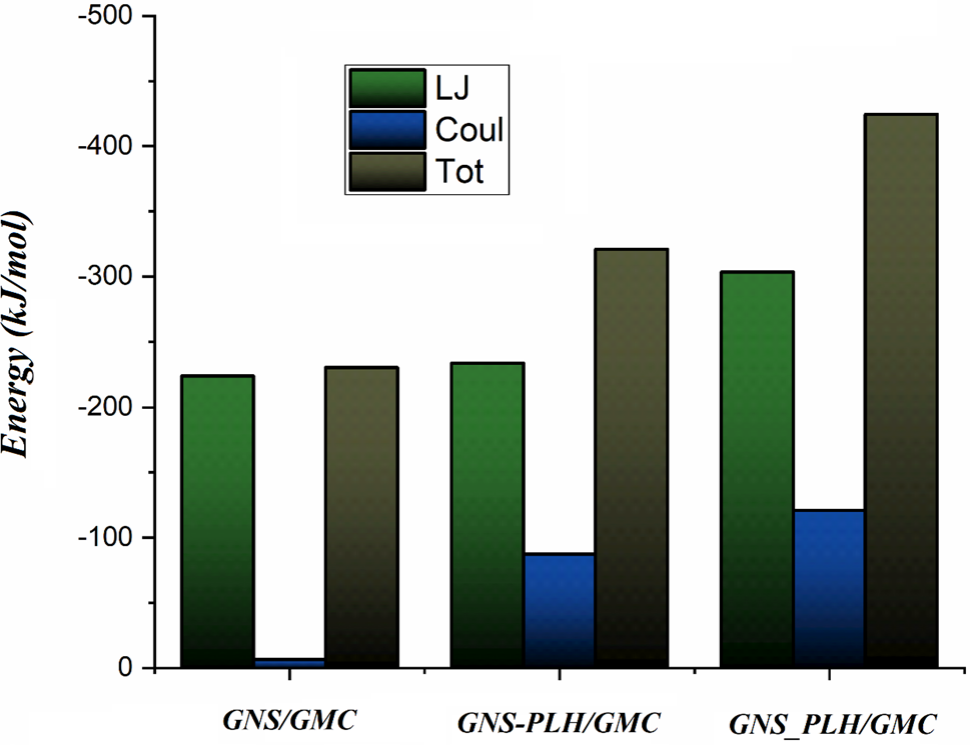
Average Lenard- Jones (Green), coulombic (Blue), and total (Brown) interaction energies between different components of loading system.

**Figure 4:**
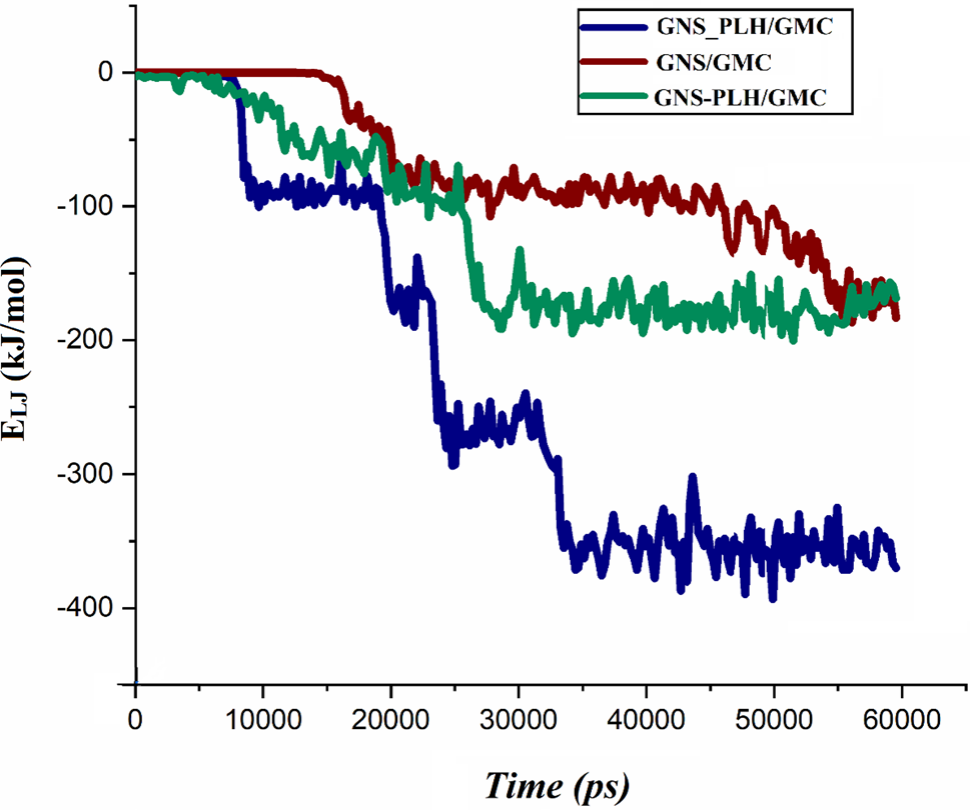
The L–J interaction energy between the GMC molecules and GNSs as a function of simulation time.

To obtain a better understanding of the GMC adsorption mechanism on the GNSs surfaces, the interaction energy is calculated. Figure 3 represents the Lenard–Jones (L–J), coulombic (Coul), and, total energies for the adsorption process. The comparison of the energy values between the three studied systems shows that in the GNS_PLH/GMC system, the contribution of L–J energy increases significantly. But in the GNS/GMC and GNS-PLH/GMC systems, L–J energy values are almost equal. This result shows that the Coul energy between drug and PLH in the GNS_PLH/GMC system increases the total energy. It seems that the Coul energy has a significant role in strengthening the total energy so that in the GNS_PLH/GMC system, it also strengthens the total energy. As illustrated in Fig. 4, L–J interactions gradually increase as more and more drugs are being adsorbed on the carrier. Close inspection of Fig. 3 shows that the total interaction energy between GMC and GNS has negative values, in the following order: GNS_PLH/GMC > GNS-PLH/GMC > GNS/GMC. This result indicates that the total energy values for the interaction of the GMC with the GNSs in F-GNSs are more than GNS, especially in the GNS_PLH/GMC system where the GNS are covalently functionalized with the PLH groups. These findings have good agreement with the depicted snapshots. The more negative total energy in this system can be brought up as witnesses for the adsorption of a more GMC (cf. 70%) on the GNS_PLH surface. Also, these results confirm that covalently functionalization GNS with PLH groups leads to the formation of a drug-carrier complex with more stability. To clearly show how the interaction energy affects the adsorption of drug molecules, all energy values are normalized. These values are calculated by dividing the total interaction energy by the number of adsorption drug molecules and results are given in Fig. S2. As shown in Fig. 2, at the end of the simulation, the GNS_PLH/GMC system had the greatest average number of adsorbed drug molecules. While the lowest average number of adsorbed drug molecules is related to the GNS/GMC system. Accordingly, the number of adsorbed drug molecules in the studied systems was consistent with the normalized energy results (see Fig. S2). The L–J, Coul, and, total energy values for the interaction of GEM with PLH in the GNS-PLH/GMC system are reported in Table S1. The energy values in this table show that the value of the total interaction energy for the GNS-GEM combination is significantly higher than the GEM-PLH pair, which shows the drug molecule tends to make stronger interactions with the GNS.

#### RDF analysis

**Figure 5:**
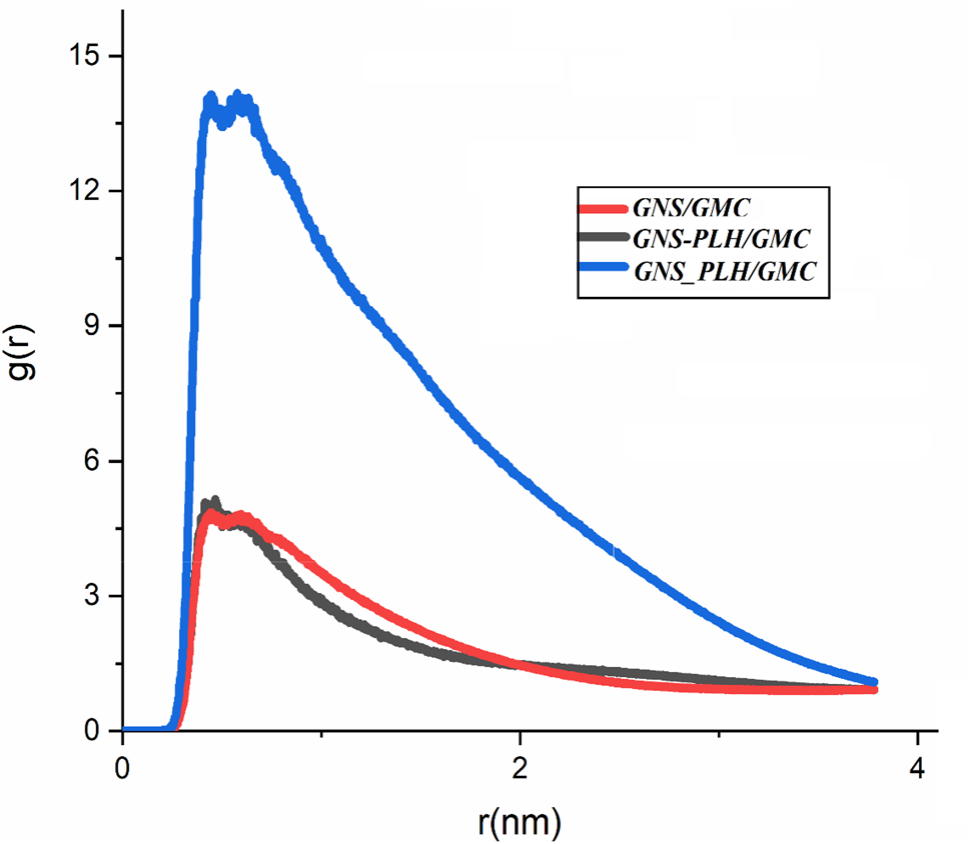
The RDF of the GMC anti-cancer drug loading onto GNS and F-GNS over 60 ns MD trajectories.

For further structural analysis consistent with our findings, the effect of PLH functional groups on the GMC adsorption is examined by the RDF analysis, and the obtained results are depicted in Fig. 5. The RDF peak of drug molecules and GNS appeared at a distance of about 0.2 nm. RDF results show obvious peaks in the distance of about 0.4 nm for the GNS/GMC and GNS-PLH/GMC systems. As expected, the increase in the intensity of the RDF peak is more pronounced for the GNS_PLH/GMC system, and its height peak is estimated at around ~ 14. These results support our arguments about the most favorable interactions of GMC with nano-carrier, resulting in higher adsorption efficiency drug on the GNS_PLH surface. In conclusion, it is predicted that GNS_PLH can carry the GMC drug most effectively, and it can be considered the most appropriate drug carrier. The lower intensity of the GNS-drug RDF peak for the GNS/GMC system confirmed the weaker interactions of the drug with the pristine nano-carrier.

#### HB analysis

**Figure 6:**
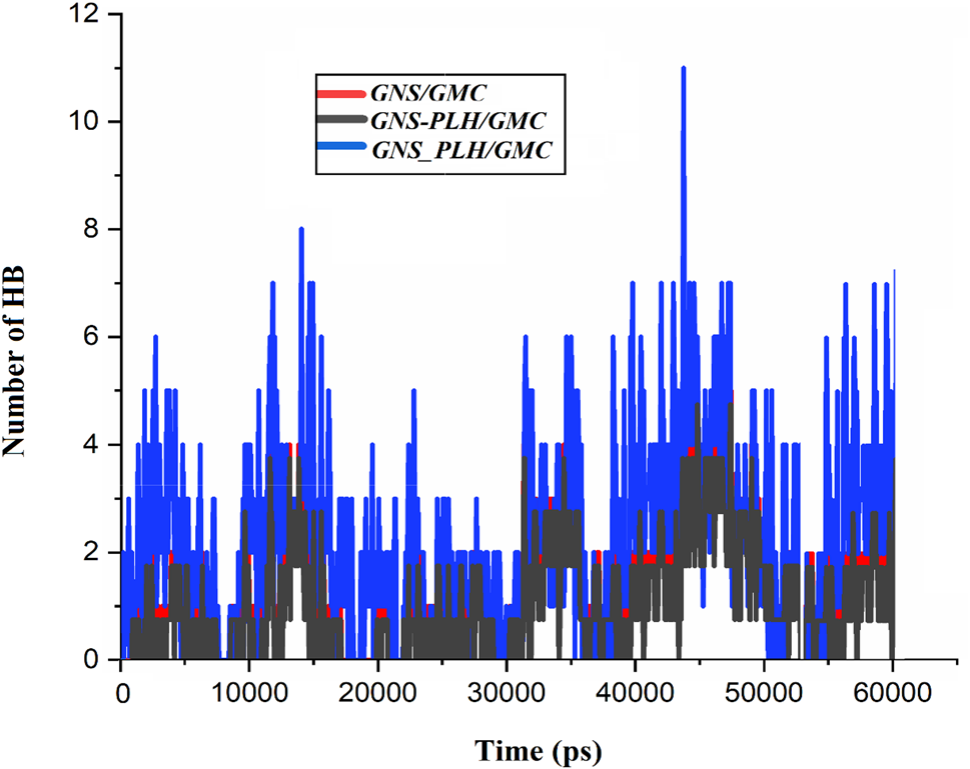
The number of HB formed between the drug and carrier.

To obtain a deep insight into the changes in the hydrogen bonding pattern, the number of HB for the intended components is calculated via the “gmx hbond” module in the GROMACS program. The HB analysis between the chosen pairs of carrier and drug in the studied complexes is carried out based on a donor–acceptor cutoff distance of 3.5 Å. Figure 6 depicts the variation in HBs formation between the carrier and the drug during simulation time. As shown in this figure, the number of HBs of the carrier-drug in the functionalized systems is more than the pristine ones, particularly in GNS_PLH/GMC system. In the other words, with the functionalization of the GNS surface, the number of host–guest HBs increased. As well as, it should be noted that the GNS/GMC system has the lowest number of HBs. This is due to the fact that in this system only four GMC molecules are adsorbed on the GNS surface, and the other GMC molecules do not tend to interact with the GNS surface or overlap with each other. Furthermore, it is clear that after adsorption of GMC on the GNS surfaces, the number of HB between drug and water molecules decreases, while the HB number between the GMC molecules and the GNS surfaces increases by passing the time (see Fig. S3). Thus, it can be concluded that hydrogen bonding can be a driving force for the adsorption of GMC molecules on the GNS and F-GNS surfaces.

#### MSD analysis

**Figure 7:**
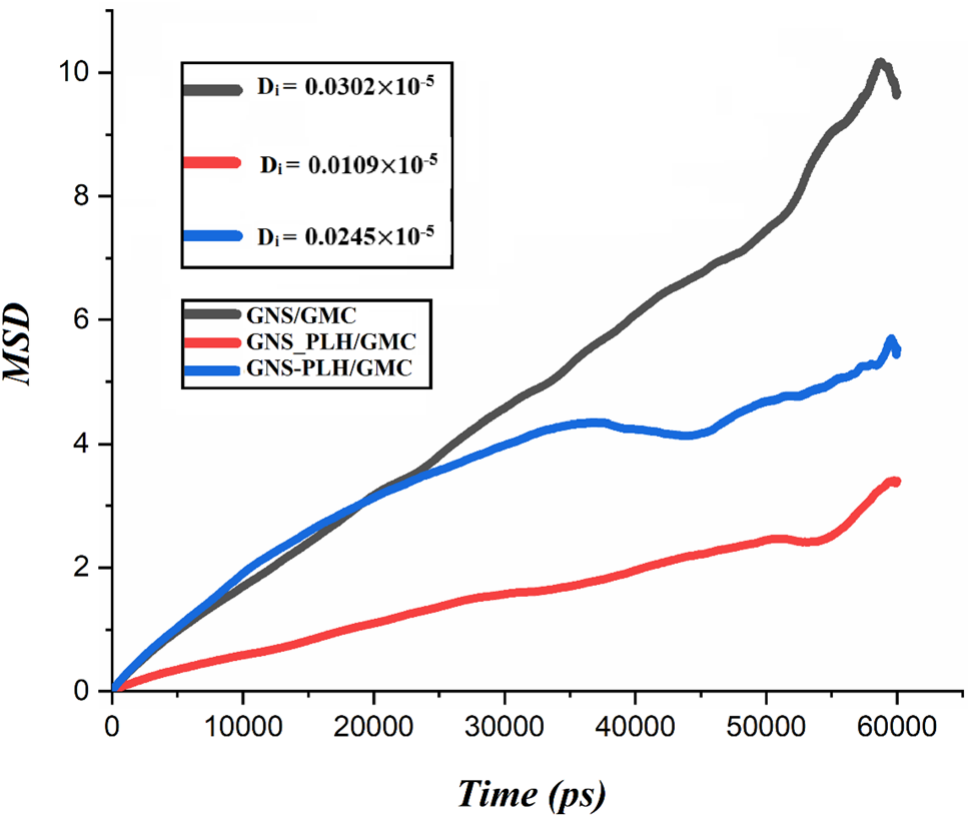
The MSD of drug molecules with carrier and corresponding D_i_ values in the investigated systems.

The D_i_ is a measure of diffusion mobility and translational of each molecule at thermodynamic equilibrium^56,57^. Figure 7 exhibits the MSDs and D_i_ of GMC molecules in three studied systems. By referring to this figure, it is found that the GNS_PLH/GMC system reveals the lowest MSD for the GMC diffusion, the GNS-PLH/GMC system affords medium drug diffusion and the GNS/GMC system has the highest one. This means that the covalent functionalization of GNS increases the interaction between a drug and the nanocarrier. For effective delivery of GMC, the system must reveal the lowest MSD, therefore, the GNS_PLH/GMC system, which possesses the slowest and the most controlled GMC transport/diffusion, can be considered the most favorable system. The lowest diffusion coefficient is observed for the GNS_PLH/GMC system (0.0109 × 10^–5^ cm^2^/s), and the GNS/GMC system shows the highest GMC diffusion coefficient (0.0302 × 10^–5^ cm^2^/s). As a result, the capacity of the DDSs to deliver the GMC obeys the following order, GNS_PLH/GMC < GNS-PLH/GMC < GNS/GMC. This fact can be related to the weaker interaction of GMC with the GNS surface. It is confirmed by the RDF plots presented in Fig. 5 that show the distance between GMC and carrier in GNS_PLH/GMC system is shorter than in the other two systems. We also calculate the diffusion coefficient of drug molecules adsorbed on the GNSs (see Table S2). According to the data in this table, the lowest D_i_ for the GNS_PLH/GMC system confirms the stronger interactions in this system. It should be noted that the highest D_i_ of GMC belongs to the GNS/GMC system which has the lowest interaction energy.

### WT-MD simulations

**Figure 8:**
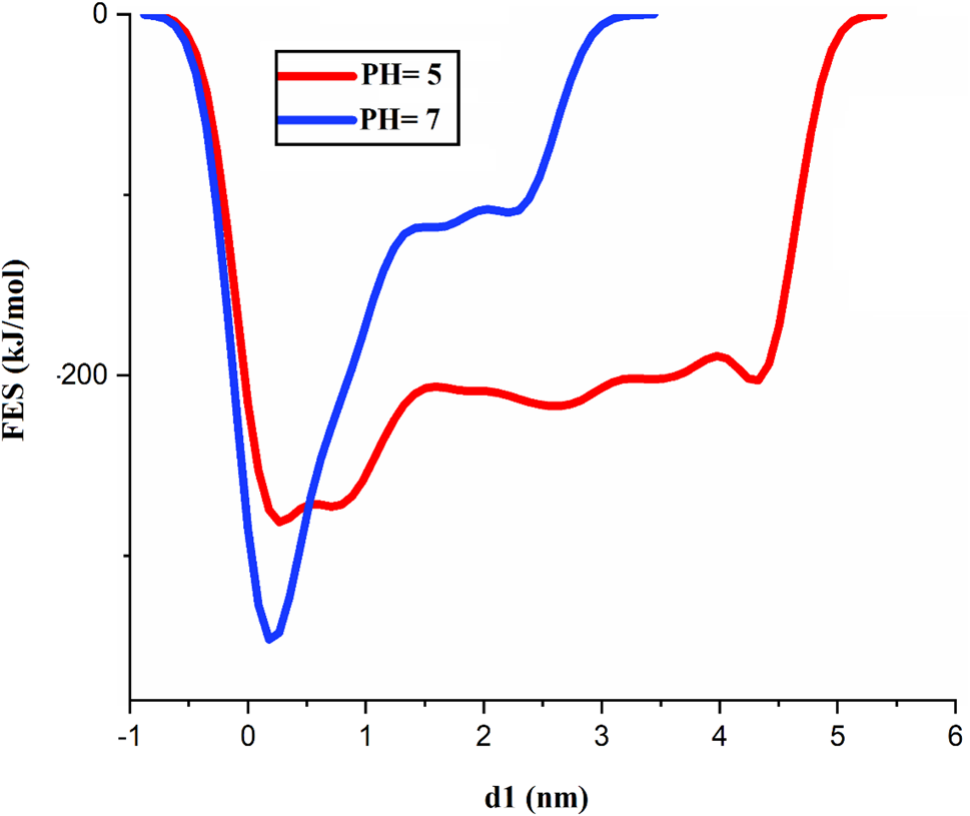
Free energy curves for the adsorption of GMC on GNSs.

The effect of functional groups on GMC loading and releasing is studied by evaluating the FE surface. In this part, the variation in FE surface relative to changes in distances between the COMs of drug and polymer, both in neutral and acidic circumstances is investigated. As shown in Fig. 8, the global minimums of GMC adsorption at neutral circumstances are set 0.17 nm away from the PLH’s COM with the release of − 346.24 kJ/mol of energy. Nevertheless, the energy release is increased by − 64.98 kJ/mol in acidic conditions. In addition, the difference between the drug FE at the time of adsorption on the polymer compared to when it is freely solved in an aqueous medium shows a significant reduction in acidic conditions. Therefore, it seems that the acidic circumstances can help to release the drug from the nanosheet.

## Conclusion

The GNS as a suitable carrier for GMC drug is investigated using the MD and WT-MD simulations, and the effect of the PLH functional group (as covalently and non-covalent modification) on the GMC adsorption is examined. The interaction energy, probability of finding the drug on the nanosheet surface, the number of HB, and mean square displacement after drug adsorption are evaluated. Assessing the interaction energies of GMC with the GNSs in the absence and presence of the polymer showed that L–J interactions have a considerable role in the GMC adsorption process. Close inspection of the FE surface of GMC revealed that the global minimum of drug adsorption at a neutral environment is set 0.17 nm away from the PLH’s COM, with a release of − 346.24 kJ/mol of energy. Whereas, in an acidic environment, the energy desorption is increased and set at − 281.26 kJ/mol. The obtained results show that the GNS_PLH due to the strong interaction with the GMC drug is an appropriate candidate for the targeted drug delivery. This result is nicely confirmed by RDF and MSD analyzes. The present work emphasizes that the GNS_PLH is an appropriate substrate for the adsorption of GMC molecules and will encourage the researcher and experimentalists to explore and use this substrate as a GMC carrier.

## Supplementary Information

Supplementary Information.

## Data Availability

The datasets used and/or analyzed during the current study available from the corresponding author on reasonable request.

## Code availability

The codes that support the findings of this study are available from the corresponding authors upon reasonable request.
