# Supplementary material for: Surface functionalization of graphene nanosheet with poly (l-histidine) and its application in drug delivery: covalent vs non-covalent approaches
Source: Sci Rep. 2022 Nov 9;12:19046. doi: 10.1038/s41598-022-21619-0 (PMC9646737; doi:10.1038/s41598-022-21619-0)
Supplement: Supplementary file 1 — Supplementary Information. [file 41598_2022_21619_MOESM1_ESM.docx]

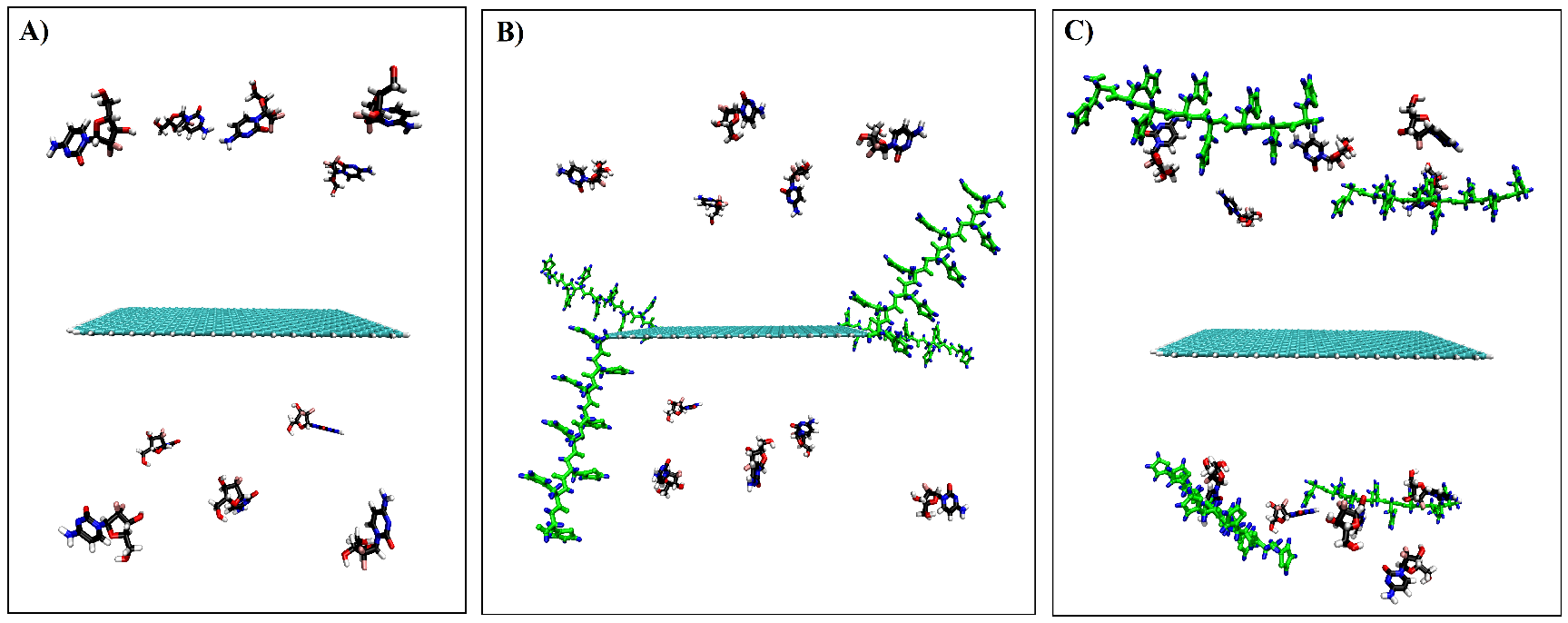


**Figure S1.** The initial snapshots of A) GNS/GMC, B) GNS_PLH/GMC, and C)

GNS-PLH/GMC systems.


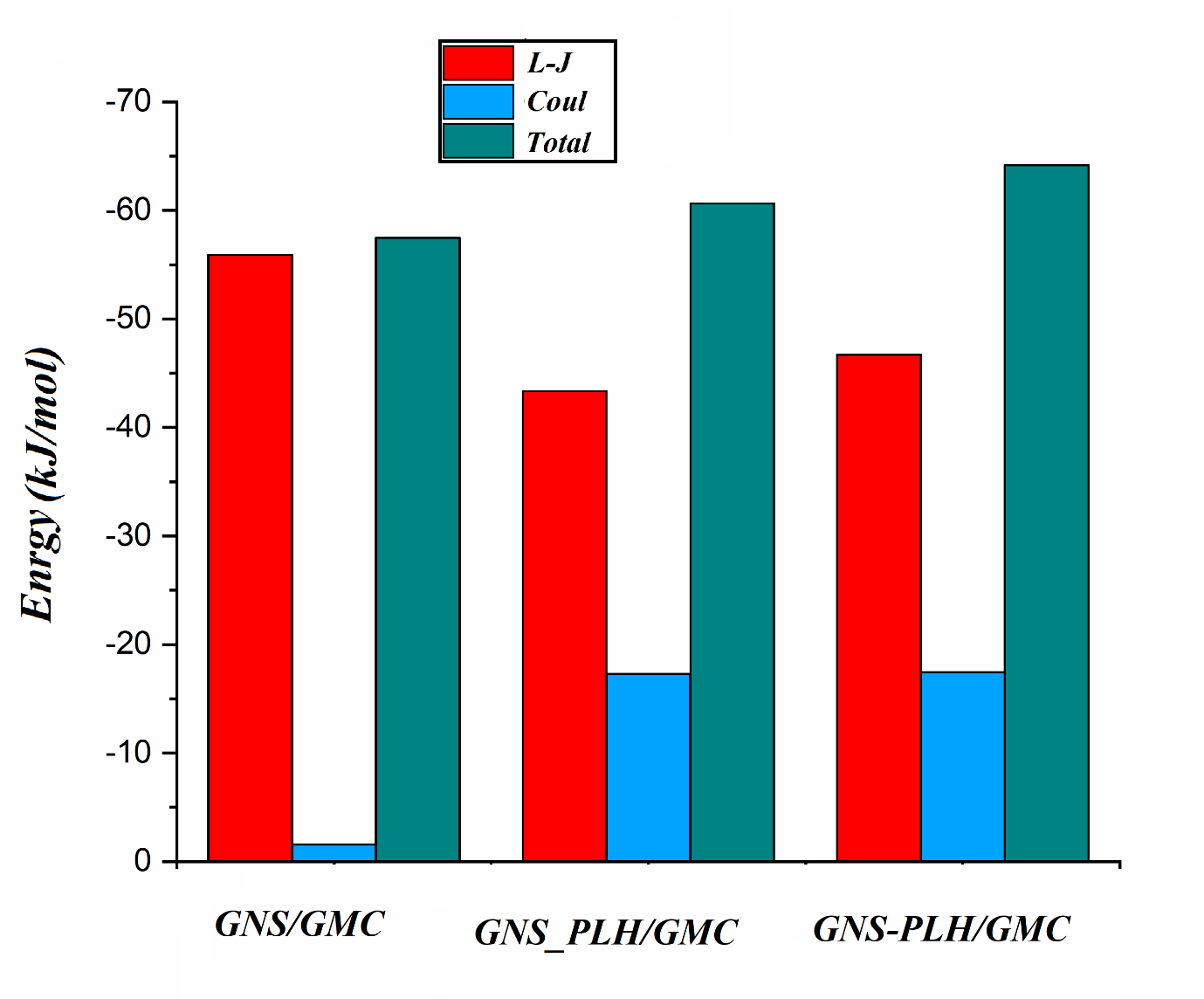
**Figure** **S2**. Normalized L-J (red), Coul (blue), and total (dark cyan) interaction energies between different components of loading system.


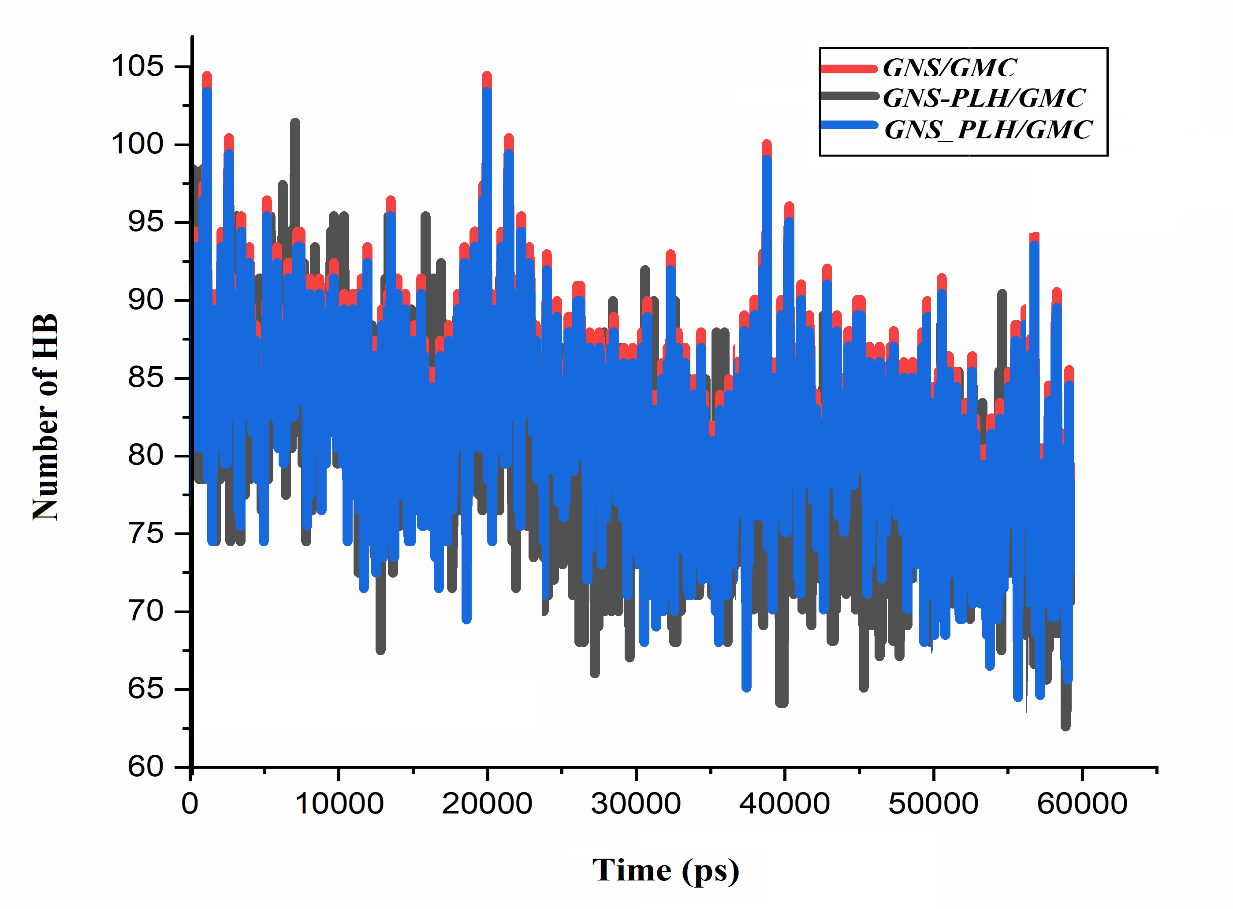


**Figure** **S3.** The number of HB formed between the drug and water.

**Table S1.** The LJ, coul, and Total energies between GMC and PLH (all in kJ/mol)

| System | LJ | Coul | Tot |
| --- | --- | --- | --- |
| GNS-PLH/GMC | -141.02 | -52.49 | -193.51 |

**Table S2.** The self-diffusion coefficient Di for the drug molecules adsorbed in the studied systems.

| Systems | D_i_ (10^-5^ cm^2^/s) | Error |
| --- | --- | --- |
| GNS/GMC | 0.027 | ±0.026 |
| GNS-PLH/GMC | 0.011 | ±0.0004 |
| GNS_PLH/GMC | 0.001 | ±0.0013 |
